# Constructing new-generation ion exchange membranes under confinement regime

**DOI:** 10.1093/nsr/nwae439

**Published:** 2024-11-29

**Authors:** Xingya Li, Peipei Zuo, Xiaolin Ge, Zhengjin Yang, Tongwen Xu

**Affiliations:** Key Laboratory of Precision and Intelligent Chemistry, School of Chemistry and Materials Science, University of Science and Technology of China, Hefei 230026, China; Key Laboratory of Precision and Intelligent Chemistry, School of Chemistry and Materials Science, University of Science and Technology of China, Hefei 230026, China; Key Laboratory of Precision and Intelligent Chemistry, School of Chemistry and Materials Science, University of Science and Technology of China, Hefei 230026, China; Key Laboratory of Precision and Intelligent Chemistry, School of Chemistry and Materials Science, University of Science and Technology of China, Hefei 230026, China; Key Laboratory of Precision and Intelligent Chemistry, School of Chemistry and Materials Science, University of Science and Technology of China, Hefei 230026, China

**Keywords:** ion exchange membranes, microporous confinement regime, ion permeability/conductivity, ion selectivity, energy storage and production

## Abstract

Ion exchange membranes (IEMs) enable fast and selective ion transport and the partition of electrode reactions, playing an important role in the fields of precise ion separation, renewable energy storage and conversion, and clean energy production. Traditional IEMs form ion channels at the nanometer-scale via the assembly of flexible polymeric chains, which are trapped in the permeability/conductivity and selectivity trade-off dilemma due to a high swelling propensity. New-generation IEMs have shown great potential to break this intrinsic limitation by using microporous framework channels for ion transport under a confinement regime. In this Review, we first describe the fundamental principles of ion transport in charged channels from nanometer to sub-nanometer scale. Then, we focus on the construction of new-generation IEMs and highlight the microporous confinement effects from sub-2-nm to sub-1-nm and further to ultra-micropores. The enhanced ion transport properties brought by the intense size sieving and channel interaction are elucidated, and the corresponding applications including lithium separation, flow battery, water electrolysis, and ammonia synthesis are introduced. Finally, we prospect the future development of new-generation IEMs with respect to the intricate microstructure observation, *in-situ* ion transport visualization, and large-scale membrane fabrication.

## Teaser

New-generation ion exchange membranes allow ion transport under a microporous confinement regime and bring about atypical ion transport behaviors, generating high ion permeability and selectivity, and thus enabling outstanding performance in ion separation, clean energy production and renewable energy storage.

## INTRODUCTION

**Figure 1.**
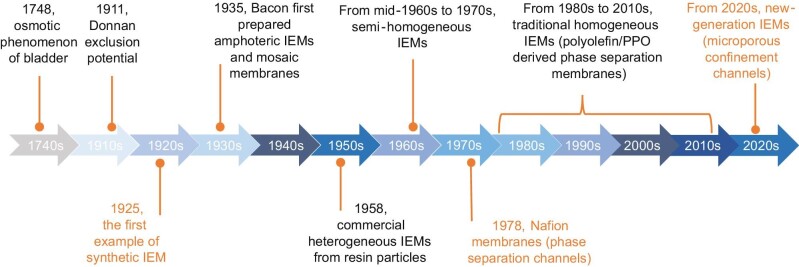
The evolution history of IEMs. Three milestones throughout the development of IEMs (i.e. the original IEMs synthesized in 1925, Nafion membranes appeared in 1978, and the emerging of new-generation IEMs in 2020). Adapted from Ref [6], WILEY-VCH GmbH.

Ion exchange membranes (IEMs) are the core component of electro-membrane processes, including electrodialysis, flow battery, water electrolysis, and ammonia synthesis via electrochemistry, demonstrating tremendous potential for precise separation, energy storage and conversion, and carbon emission reduction [1,2]. The major function of IEMs (i.e. cation exchange membranes and anion exchange membranes) is the fast and selective ion transport and the partition of anode and cathode reactions [3]. The ion permeability/conductivity and selectivity of IEMs are dependent on the ion transport properties in the charged channels of membranes, which determine the efficiency of the electro-membrane processes [4,5]. Three milestones are noteworthy throughout the evolution history of IEMs. The first one is the original IEMs synthesized in 1925, and Nafion membranes well-known for the phase separation channels appeared in 1978 [6] (Fig. 1). The emergence of new-generation IEMs began in 2020, and featured microporous framework channels with an ion confinement region, where the transported ions interact with the channel wall to deliver a fast and selective transport.

**Figure 2.**
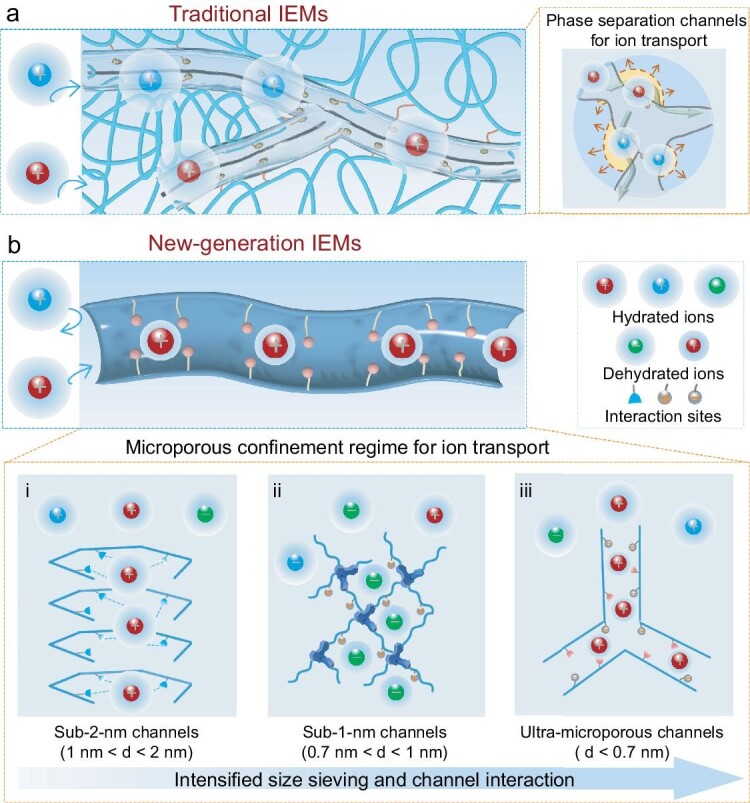
The structures of ion exchange membranes (IEMs). (a) The structure of conventional IEMs. The ion transport channels are at the nanometer-scale formed by hydrophobic and hydrophilic phase separation. Ions transport across the membrane through exchange with charged groups. IEMs are composed of flexible polymer chains forming ion channels with a high swelling propensity. (b) The structure of new-generation IEMs under a microporous confinement regime. (i) Sub-2-nm channels (i.e. 1 nm < d < 2 nm). (ii) Sub-1-nm channels (i.e. 0.7 nm < d < 1 nm). (iii) Ultra-microporous channels (i.e. d < 0.7 nm). The microporous channels are formed by the specific staking of polymer chains with sizes ranging from sub-2-nm to sub-1-nm and further to sub-0.7 nm, where the size sieving and channel interaction are intensified, generating atypical ion transport behaviors.

Traditional IEMs form ion transport channels through hydrophobic and hydrophilic phase separation. The charged channels are at the nanometer scale and mainly distributed in the hydrophilic region with ion exchange groups, whereas the hydrophobic region is devoid of a porous structure for ion transport [6] (Fig. 2a). The ion flux of IEMs is positively correlated to the amount of ion exchange sites (i.e. ion exchange capacity, IEC) of the charged channels. The counter-ion flux can be increased and the co-ion flux can be suppressed via enhancing the IEC, leading to an improved selectivity for counter-ions over co-ions. However, a dramatic increase of IEC can lead to the swelling of IEMs composed of flexible polymer chains that generate ion channels with a much larger size, which can hardly enable a selectivity among ions of the same charge. Therefore, traditional IEMs endlessly encounter a challenge regarding the trade-off between ion flux and selectivity.

New-generation IEMs refer to the membranes that combine functional groups and rigid micropore confinement with a customized chemical environment for specific ions. Ion transport therefore hinges on the synergistic effect of the confined size effect and the interaction between ions and channel walls. New-generation IEMs are constructed under the guidance of micropore-confined ion transport effects, aimed at breaking through the trade-off limitation of conventional IEMs (Fig. 2b). The confined channels with a size comparable to the hydrated ion diameter can induce a dehydration of ions. The dehydrated ions expose more intrinsic chemistry to interact with the channel wall, generating a stronger interaction between ions and the channel wall. The size sieving and interaction screening effects under a microporous confinement regime are prominently reinforced and dominate the ion transport properties (Fig. 2b–i, ii, iii). New-generation IEMs have demonstrated great potential to break the inherent limitation by employing rigid microporous framework channels with confinement regime for ion transport, which are expected to become a substitute for conventional IEMs in the electro-membrane processes.

This review starts with the description of the fundamental principles of ion transport in the charged channels at a scale from ∼100 nm to sub-1-nm. Then, we introduce the challenges of traditional IEMs with charged channels at the nanometer scale, such as two-phase IEMs and three-phase IEMs, where ions transport via exchange with charged groups. We highlight the new-generation IEMs, constructed from microporous confined ion channels with sizes ranging from sub-2-nm to sub-1-nm and further to ultra-micropore. The enhanced ion transport behaviors brought by the intensified size sieving and channel interaction under rigid microporous confinement are analyzed, followed by the illustration of their applications including Li^+^ selective separation, flow battery, water electrolysis, and ammonia synthesis. Finally, the outlook and perspectives regarding new-generation IEMs under a confinement regime are proposed.

## ION TRANSPORT WITHIN CHARGED CHANNEL

**Figure 3.**
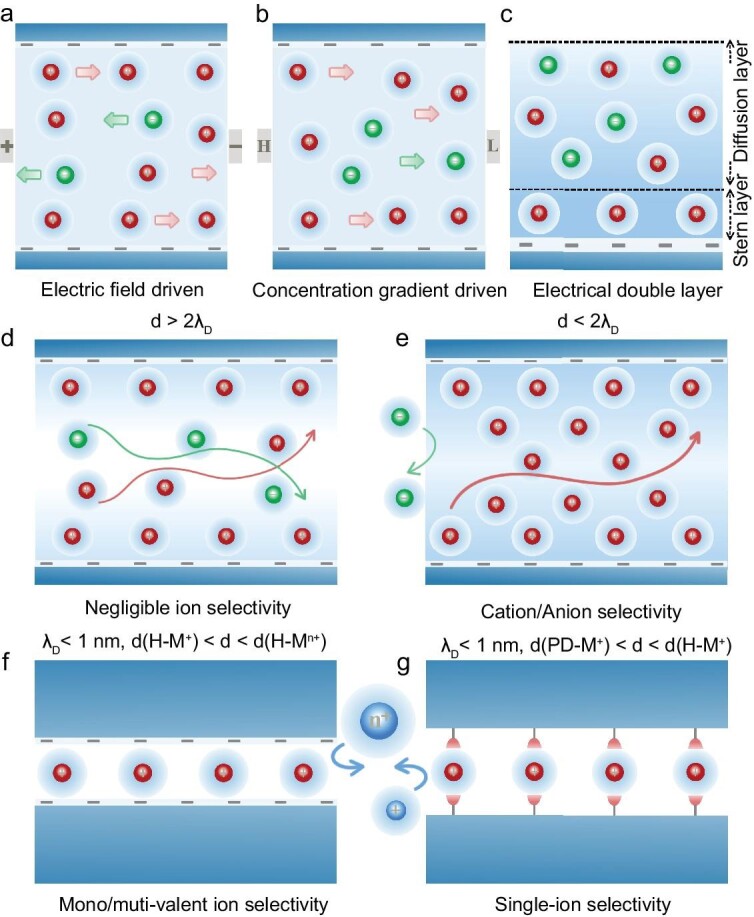
Ion transport behaviors in charged channels. Ion transport in the charged channels driven by (a) the electric field and (b) the concentration gradient, where the ion transport behaviors can be depicted by the Poisson and Nernst-Planck equations. (c) The formation of an electrical double layer in the charged channels. The distance between the two layers is termed as the Debye length (λ_D_). (d) d > 2 λ_D_: similar cation and anion flux, but no ion selectivity. (e) d < 2 λ_D_: higher cation flux than anion flux, and high cation/anion selectivity. (f) λ_D_ < 1 nm, d(H-M^+^) < d < d(H-M^n+^): higher monovalent cation flux than multivalent cation flux, and high mono-/multi-valent cation selectivity. Monovalent ions undergo a partial dehydration with a lower energy barrier than multivalent ions. (g) λ_D_ < 1 nm, (d(PD-M^+^)) < d < d(H-M^+^): higher specific monovalent cation flux than other monovalent cation flux, and single monovalent cation selectivity. Monovalent ions undergo a partial dehydration and the specific ion interaction enables a single ion selectivity.

In the bulk solution, ion transport involves three scenarios, including fluid convection, electrical migration, and concentration gradient diffusion [7]. In the charged channels, the fluid velocity can be ignored, where ions are driven by the electric field (Fig. 3a) and the concentration gradient [8] (Fig. 3b). The ion transport behaviors are associated with the electrical potential and local concentration, which can be described by the Nernst-Planck (N-P) equation (1) [9]. In order to accurately depict the ion transport process in the charged channels, besides the effect of the electric field on ions, the adverse effect of the concentration difference on the electric field should be taken into consideration. Therefore, the Poisson equation (2) is coupled with the N-P equation under certain boundary conditions to give a general description of ion transportation [10].


(1) \begin{eqnarray*}
{{\overline J }_i} = - {{D}_i}\Delta {{c}_i} - \frac{{{{z}_i}F}}{{RT}}{{D}_i}{{c}_i}\Delta \Phi ,
\end{eqnarray*}

(2) \begin{eqnarray*}
{{\Delta }^2}\Phi = - \frac{e}{\varepsilon }({{c}_ + } - {{c}_ - }),
\end{eqnarray*}

where *J_i_, D_i_, c_i_, z_i_*, and Φ are the ion flux, diffusion coefficient, ion concentration, valence of ionic species, and chemical potential, respectively. *F, R, T, e*, and *ε* are the Faraday constant, universal gas constant, Kelvin temperature, electron charge, and dielectric constant, respectively.

The charges on the channel wall can adsorb counter-ions forming an electrical double layer (EDL) [11] (Fig. 3c). Specifically, in a negatively charged channel, counter-ions (i.e. cations) can be adsorbed while co-ions are excluded (i.e. anions), and vice versa in a positively charged channel. The adsorbed cations form a dense layer designated as the Stern layer, due to the strong electrostatic attraction toward cations and repulsion toward anions exerted by the negatively charged channel wall. Next to the Stern layer is the starting boundary of the relatively loose diffusion layer, comprised of more cations and fewer anions. The end boundary of the diffusion layer shares a similar composition to the bulk solution. The distance of the two layers is termed as the Debye length (*λ_D_*), representing the effective distance of the charged surface and normally ranges from 1 to 100 nm [12]; *λ_D_* is related to the ionic species, ion concentrations, and pH values of the solution [13] and can be calculated from equation (3):


(3) \begin{eqnarray*}
{{\lambda }_D} = \sqrt {\frac{{{{\varepsilon }_r}{{\varepsilon }_0}kT}}{{2{{C}_{{bulk}}}{{z}^2}{{e}^2}}}},
\end{eqnarray*}

where *ε_r_, ε*_0_, *k, T, C_bulk_, z*, and *e* are the relative permittivity of the solution, permittivity of a vacuum, Boltzmann constant, Kelvin temperature, concentration of the bulk solution, valence of ionic species, and electron charge, respectively.

The ion transport behaviors (e.g. ion flux and ion selectivity) are prominently influenced by the relationship between the channel diameter (d) and the Debye length [8,14]. When the channel diameter is much larger than the Debye length (i.e. d >2 λ_D_) (Fig. 3d), the EDLs are shielded by the bulk solution phase in the channel, and ions transporting in the channels are similar to those in the bulk solution. The covered EDL can hardly affect the transport process of either cations or anions independent of the charges of the channel wall. Thus, negligible ion selectivity can be observed. When the EDL starts to overlap (i.e. d <2 λ_D_) (Fig. 3e), the EDLs are exposed by the depletion of the bulk solution phase in the channel, attracting the counter-ions to accumulate in the charged channels. For instance, if the channel wall is negatively charged, the EDL can promote cation transport and inhibit anion transport via the accumulation of cations and depletion of anions, respectively, leading to a selectivity for cations over anions. Likewise, the positively charged channels are selective for anions over cations.

As the Debye length decreases to sub-1-nm, where the channel size approaches the diameter of hydrated ions, the space confined effects become dominant in determining the ion transport behaviors. For monovalent and multivalent ions, they should undergo a partial dehydration to pass through the channel, since the channel size is larger than the diameter of hydrated monovalent ions (d(H-M^+^)) but smaller than that of the hydrated multivalent ions (d(H-M^n+^)). Smaller monovalent cations overcome a lower energy barrier than that of larger multivalent cations based on the confined size sieving effect, so monovalent cations transport faster than multivalent cations, leading to a higher ion flux and selectivity for monovalent cations rather than multivalent cations. Specifically, the multivalent cations can be referred to divalent and trivalent cations. According to the energy barrier passing through the channel, the ion flux follows the order of: monovalent cations > divalent cations > trivalent cations and the ion selectivity follows the same sequence [4] (Fig. 3f). For monovalent ions of similar sizes, when the channel size is smaller than the diameter of hydrated monovalent ions (d(H-M^+^)) but larger than the diameter of partially dehydrated monovalent ions (d(PD-M^+^)), the ion's chemistry can be exposed by further dehydration to form a specific interaction between monovalent ions and the channel wall, generating a single-ion selectivity among monovalent ions according to the confined interaction screening effect and only the specific ion can pass through the channel (Fig. 3g). Moreover, the specific ion-channel interaction can facilitate ion transport by compensating for the dehydration energy barrier [15]. If the channel is positively charged, the transport behaviors of anions are similar to those of the cations in negatively charged channels.

## TRADITIONAL ION EXCHANGE MEMBRANES AND ION TRANSPORT THEREIN

Traditional ion exchange membranes (IEMs) possess a microphase-separated structure, where the hydrophobic phase is nonporous and blocks the transport of ions, and the hydrophilic phase generates nanochannels with charged sites for ion transport via ion exchange [16]. The Donnan-Manning model is applied to describe ion transport properties in the charged nanochannels within traditional IEMs. Ion mobility is primarily affected by the tortuosity of the nanochannel and electrostatic effect, simulated by the Mackie–Meares model [17] and Manning's counter-ion condensation theory [18,19], respectively. The ion mobility in IEMs is represented by Manning's equation (4) [19], and the ion selectivity of the IEMs can be calculated with equation (5):


(4) \begin{eqnarray*}
D_i^m = D_i^s{{f}_{u,i}}{{\varphi }_w}{{\left(\frac{{{{\varphi }_w}}}{{2 - {{\varphi }_w}}}\right)}^2}\left(1 - \frac{{z_i^2}}{3}A\right),
\end{eqnarray*}

(5) \begin{eqnarray*}
S_{{i / j}}^u = \frac{{{{z}_i}D_i^s}}{{{{z}_j}D_j^s}}\frac{{{{f}_{u,i}}}}{{{{f}_{u,j}}}}\frac{{\big(1 - \frac{{z_i^2}}{3}A\big)}}{{\big(1 - \frac{{z_j^2}}{3}A\big)}},
\end{eqnarray*}

where *D_i_^m^* is the ion diffusion coefficient inside the membrane, and *D_i_^s^* is the ion diffusion coefficient in the solution. *f_u,i_* is the fraction of counter-ion inside the membrane without a condensed state. *φ_w_* is water volume fraction, and (*φ_w_*/2−*φ_w_*)^2^ represents the tortuosity degree. Parameter *A* represents the electrostatic effect.

**Figure 4.**
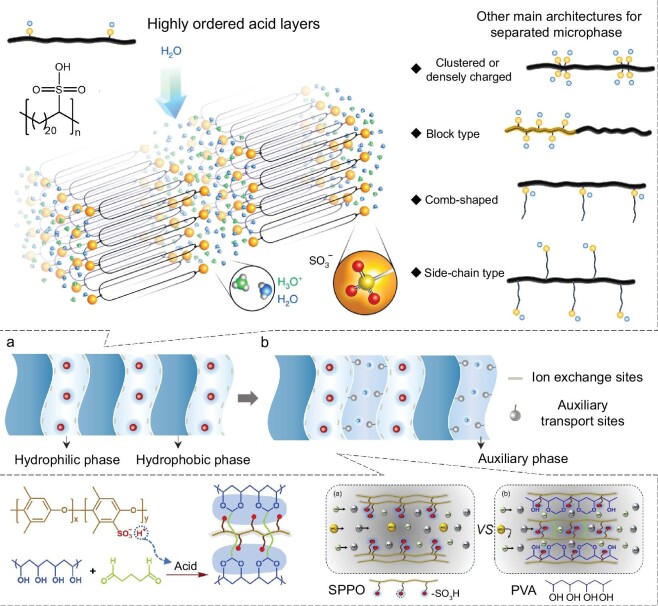
The channel structure of traditional IEMs. (a) Two-phase IEMs with hydrophobic and hydrophilic phases. Ions can only transport in the hydrophilic phase. A typical example is the precisely sulfonated polyethylene with highly ordered acid layers (top left corner). Adapted from Ref [21], Springer Nature Limited. Other common polymer architectures for enhancing microphase-separated structures are also shown. Adapted from Ref [2], John Wiley and Sons. (b) Three-phase IEMs with hydrophobic, hydrophilic, and auxiliary phases. Ions can transport in the hydrophilic and auxiliary phases. A typical example is shown, that is, introducing polyvinyl alcohol as an auxiliary phase within sulfonated polyphenylene oxide membranes. Adapted from Ref [23], Elsevier.

Two-phase IEMs are first discussed as an example, which consist of hydrocarbon-based polymeric backbones as the hydrophobic region and side chains with charged groups as the hydrophilic region [20] (Fig. 4a). The hydrophobic phase has a dense structure posing an extremely high energy barrier for ions to pass through. The hydrophilic phase can form charged channels via the self-assembly of side chains with charged sites during the microphase separation process. Ion transport behaviors in the hydrophilic charged nanochannels of the two-phase IEMs are controlled by the EDL as previously discussed. The two-phase IEMs show a cation/anion selectivity by conducting counter-ions and blocking co-ions through electrostatic interactions, which is consistent with the Donnan-Manning model. However, a selectivity for ions of the same charge can hardly be observed for the two-phase IEMs. The ion flux is dependent on the ion exchange capacity (IEC) of the hydrophilic charged channels. With increasing ion exchange capacity, the fluxes of counter-ions increase, while the co-ions fluxes are suppressed. From the above discussion we can see that the selective ion transport through these IEMs hinges largely on the hydrophilic regions, and thus most studies on high-performance two-phase IEMs focus on strategies that can enhance the self-assembly of charged polymers into microphase-separated structures and for this purpose, varied polymer architectures have been developed, for example precisely sulfonated polyethylene with highly ordered acid layers [21]. Other typical architectures include clustered or densely charged type, block type, comb-shaped type, and side-chain type, etc. [2,22] (Fig. 4a).

Some industrial electro-membrane processes, such as diffusion dialysis and electrodialysis for acid or base recovery, require IEMs capable of conducting proton or hydroxyl ions but blocking metal ions or other large anions. Under this background, three-phase IEMs used exclusively for electrodialysis and diffusion dialysis have been developed through the introduction of additional auxiliary segments [23] (Fig. 4b). An additional auxiliary region is introduced to construct three-phase IEMs, so as to facilitate the transport of co-ions and improve the selectivity for ions of the same charge [24]. The auxiliary region is composed of weak acidic (–COOH) or basic (–NH_2_, –NH, –NR_2_) groups, which can form the acid-base ion pairs with protons (H^+^) or hydroxide ions (OH^−^) via hydrogen bonding interactions. In the three-phase IEMs, the auxiliary phase can provide additional pathways for H^+^ and OH^−^ ion transport with lower energy barriers and simultaneously exclude the co-ions of much larger sizes, while the conventional two-phase IEMs retain the function of conducting counter-ions and blocking co-ions. Thus, the three-phase IEMs can realize a selectivity for H^+^ or OH^−^ ions over the according co-ions. The flux of H^+^ and OH^−^ ions can be increased by enlarging the auxiliary phase, which will in turn result in the leakage of large co-ions and diminish the ion selectivity. Overall, the increased IEC can boost the adsorption of water molecules and result in swelling of the nanochannels to a much larger size, which will cause a raising in ion flux but a decline in ion selectivity.

In summary, traditional IEMs including the two-phase and three-phase IEMs possess a hydrophobic phase, which is impermeable to ions, resulting in a high energy barrier for ion transport. Besides, the polymeric backbones are flexible and tethered with side chains of hydrophilic charged groups, showing a high propensity to swell by adsorbing water molecules. As a result, traditional IEMs can hardly maintain the integrity of the channel structure under a high IEC. Inevitably, there is always a trade-off effect between the ion flux and selectivity which is challenging for traditional IEMs. Therefore, the design of new-generation membranes should increase the porosity of membranes to lower the energy barrier for ion transport while improve the rigidity of the polymeric backbone to cope with the swelling issue. More importantly, confined space effects should be involved in the new-generation IEMs to enhance the interactions between ions and channels, aiming at a high ion selectivity.

## CONSTRUCTION OF NEW-GENERATION IEMS WITH MICROPOROUS CONFINEMENT EFFECTS

Transition-state theory (TST) depicts elementary rate processes, such as molecular permeability, which can also be deployed to explain the molecular transport in membranes [15]. Based on TST, molecules transporting from one site to the next in the confined space should be thermally activated to overcome free energy barriers, where species are required to possess sufficient internal energy including kinetic and potential energy in the microscopic scale, and have an appropriate configuration state as well. Hence, the intrinsic molecular permeability (*P*) (equation (6)) can be formulated in the form of an Arrhenius-type equation [25]. Noteworthily, the core equation of TST (equation (7)) (i.e. the Eyring equation) is represented in the following form [26,27]:


(6) \begin{eqnarray*}
P = {{P}_0}\exp \left( - \frac{{{{E}_a}}}{{RT}}\right),
\end{eqnarray*}

(7) \begin{eqnarray*}
k = \kappa \frac{{{{\kappa }_B}T}}{h}\exp \left(\frac{{\Delta {{S}^\dagger }}}{{RT}}\right)\exp \left( - \frac{{\Delta {{H}^\dagger }}}{{RT}}\right),
\end{eqnarray*}

where *P*_0_ is a pre-exponential factor, *E_a_* is the free energy barrier, *T* is the absolute temperature, and *R* is the universal gas constant. *k* is a rate constant, and Δ*S*$^\dagger$ and Δ*H*$^\dagger$ are the entropy of activation and enthalpy of activation, respectively. *κ* is the transmission coefficient, *κ_B_* is the Boltzmann constant, and *h* is the Planck constant.

As a result, the pre-exponential factor and the free energy barrier within the exponent of the molecular permeability in equation (6) can be related to the entropy and enthalpy components in equation (7), respectively [28]. The molecular permeabilities within membranes are mainly dependent on the entropic and enthalpic effects [29]. At the microscopic level, entropy represents the number of accessible configuration states, that is the higher the accessible microstates or freedom of molecular motion, the lower the activation energy. For mass transport in membranes, the transmission state is limited by the steric constraint and a low entropy of species with a small size is formed. For the transport species, enthalpy represents the internal energy, consisting of both kinetic energy and potential energy. The kinetic energy, such as translation, rotation, and vibration, is supposed to be evenly distributed within the membranes for any species. The potential energy is related to the interactions between transported species and membranes, which are exerted by the chemical bonds or other physical force fields. A lower enthalpy means a higher affinity interaction between species and membranes. According to these relations, a low entropy and a low enthalpy contribute to a high membrane permeability and membrane selectivity is determined by the differences in the entropy and enthalpy of transported species [30]. Specifically, the entropic selectivity can be generated when one species encounters a higher steric hindrance than another species during transmission. Likewise, the enthalpic selectivity can be established when the membrane occupies a stronger interaction toward one species rather than another.

The kinetic barrier networks demonstrate that the energy barrier for ion partitioning of the interface between bulk solution and polymeric membrane is higher than that for ion diffusing in the membrane during the ion traversing membrane process [31]. The ion partitioning energy barrier at the interface mainly arises from the size sieving and dielectric (ion dehydration) effect. The degree of freedom decreases when ions transport from the unconfined space to the confined space, generating a declined entropy. The ion partitioning energy barrier for ions entering a microporous-confined channel is much higher than that of a nano-confined channel. Thus the ion-channel interaction, especially at the pore entrance, should be introduced into membranes of micropores, so as to lower the enthalpy by compensating the ion partitioning energy [32].

Based on the entropic and enthalpic theory and the kinetic barrier networks, two basic guidelines should be top priority for the design of new-generation IEMs under a confinement regime. First, the designed membranes should have micropores with rigid frameworks, which can reduce the energy barrier by providing pathways for ion transport and circumvent the swelling issues of charged channels. In addition, the size of the membrane channel should be comparable to the ion diameter, thereby enhancing the interaction between ions and the functional sites on the channel wall. Herein, we define the new-generation IEMs as micropore-confined IEMs that employ micropores as confined ion transport channels, where the size sieving and interaction screening effects are substantially intensified, playing a dominant role in promoting fast and selective ion transport. The preparation of typical new-generation IEMs and ion transport behaviors under a microporous confinement regime are depicted.

### Preparation of new-generation IEMs with microporous channels

According to the recent progress of IEMs, there are three main strategies for the synthesis of new-generation IEMs, including the *in-situ* crosslinking of flexible polymer chains, the ionization of polymers with intrinsic microporosity, and the construction of microporous framework polymeric membranes. These strategies enable the construction of IEMs with micropores ranging from sub-2-nm to sub-1-nm and further below 0.7 nm (ultra-micropore). In addition, channel rigidity augments with the membrane structure evolving from the crosslinking networks to the semi-rigid polymeric skeletons and to the rigid microporous polymeric frameworks, accompanied by the ion transport channels from a disordered to aligned arrangement.

**Figure 5.**
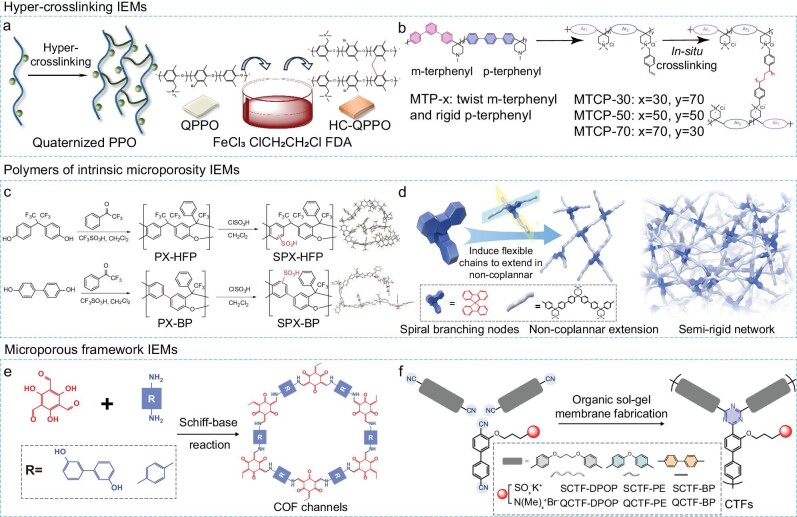
Construction of new-generation IEMs. (a) Preparation of hyper-crosslinking QPPO membranes of sub-1-nm channels. Adapted from Ref [33], John Wiley and Sons. (b) Preparation of m-terphenyl combined p-terphenyl (MTCP) membranes of ultra-microporous channels (i.e. <0.7 nm) via a superacid-catalyzed synthesis and *in-situ* crosslinking. Adapted from Ref [34], Springer Nature Limited. (c) Preparation of polymers with intrinsic microporosity (PIM) membranes of sub-1-nm channels via a superacid-catalyzed polycondensation. Adapted from Ref [36], John Wiley and Sons. (d) Superacid-catalyzed synthesis of spiro-branched poly(aryl piperidinium) (SBP) membranes of sub-1-nm channels (i.e. between 0.7 nm and 1 nm). Adapted from Ref [38], Springer Nature Limited. (e) Preparation of covalent organic framework (COF) membranes of sub-2-nm channels (i.e. between 1 nm and 2 nm) via a Schiff-base reaction. Adapted from Ref [52], John Wiley and Sons. (f) Preparation of covalent triazine framework (CTF) membranes via a superacid-catalyzed organic sol-gel route. Adapted from Ref [51], Springer Nature Limited.

First is the *in-situ* crosslinking method. Traditional IEMs have hydrocarbon backbones as the main chains and charged groups as the side chains. The flexible polymer chains can be crosslinked to form a hyper-crosslinking network with a microporous structure. For example, the linear quaternized poly(2,6-dimethyl-1,4-phenyleneoxide) (QPPO) was used as the flexible polymer chains, followed by immersion in a mixture of formaldehyde dimethylacetal (FDA), FeCl_3_, and 1,2-dichloroethane (solvent), where FDA and chloride salt served as the cross-linker and catalyst, respectively [33]. This approach can convert flexible charged polymer backbones to highly restricted networks, creating microporous channels with sizes below 0.9 nm (Fig. 5a). Another IEM was prepared by superacid catalyzed polymerization to form relatively rigid polymer chains, which were then crosslinked to the M-terphenyl combined p-terphenyl (MTCP) anion exchange membranes (AEMs) [34]. The pore size distribution of MTCP-x can be tuned by the ratios of the twist monomer to the rigid monomer. The MTCP-50 membrane displays a much narrower pore size distribution (i.e. a channel of ∼5.0 Å) as the rigid monomer content accounts for 50%, and the proportion of interconnected ultra-micropores (i.e. <0.7 nm) increase substantially (Fig. 5b). Besides, the cross-linked and partially rigid structure enables the MTCP membrane with a record-high alkaline stability.

The second strategy is the ionization of polymers with intrinsic microporosity. Polymers with intrinsic microporosity (PIM) form interconnected free volumes due to the inefficient packing of contorted chains, thereby generating continuous ion transport channels. PIMs derived IEMs are fabricated by endowing the channels with charged groups [35–37]. These charged microporous polymers are synthesized via the superacid catalyzed polycondensation of trifluoroacetophenone and aromatic diphenol monomers, followed by post-sulfonation with chlorosulfonic acid. The channel sizes of charged PIMs are at the sub-1-nm scale (i.e. between 0.5 nm and 0.9 nm) (Fig. 5c). Spiro-branched poly(aryl piperidinium) (SBP) microporous IEMs were fabricated by the combination of poly(aryl piperidinium)-based all-carbon backbone and the stereo-contorted spirocyclic unit [38]. The all-carbon backbone is prepared by superacid-catalyzed synthesis, and the semiflexible and loosely-packed network with interconnected free volumes is constructed via the joining of the stereo-contorted spirobifluorene monomers. The rigid spirobifluorene monomers serve as spiral branching nodes to connect the flexible chains in a noncoplanar direction and prevent the chains from packing, creating abundant sub-1-nm pores from 0.6 to 1 nm (Fig. 5d). Moreover, the ionic conjugated microporous polymeric (CMP) membranes display a narrow pore-size distribution around 0.7 nm [39,40]. Ionic CMP membranes are fabricated via a coelectro-polymerization strategy, where a customized monomer consisting of a carbazole moiety of electroactivity and a long ionic alkyl chain reacted with a rigid monomer (i.e. 1,3,5-tris (N-carbazolyl) benzene). The ionic alkyl chains endow the micropores of the CMP membranes with charges and a semi-rigid structure without sacrificing intrinsic pores [41].

Third is the construction of microporous framework polymeric membranes. Covalent organic frameworks (COFs) have inherent aligned channels with abundant interaction sites by the π-π stacking of aromatic cores. There are several common methods for preparing COF membranes, such as *in-situ* growth [42], nanosheet assembly [43,44], and interfacial polymerization [45–47] (Fig. 5e). A porous substrate supported COF membrane is prepared by the *in-situ* growth strategy, where a micrometer-thick COF layer is grown directly from the substrate surface. As for the nanosheet assembly approach, the COF nanosheet is first exfoliated from the bulk material, followed by vacuum filtration of the COF nanosheet suspension onto a porous substrate to form a membrane via self-assembly. In the interfacial growth process, the amine monomer and aldehyde monomer diffuse to the interface of a porous matrix and form a nanometer-thin COF membrane. Other strategies derived from the above methods are also employed to fabricate free-standing COF membranes [48,49]. The channel size of a crystalline and defect-free COF membrane is generally below sub-2 nm (e.g. between 1 nm and 2 nm). Porous aromatic framework (PAF) membranes have rigid backbones and stable C-C coupling bonds, showing excellent chemical and dimensional stability [50]. A dense and continuous PAF layer was grown onto the silicon substrate decorated with reactive sites via a liquid-solid interfacial polymerization, followed by etching the interlayer between the substrate and PAF to obtain a free-standing membrane. The channel size of the PAF membrane is also at the sub-2 nm scale. Covalent triazine framework (CTF) membranes are fabricated using aromatic nitrile monomers of functional sites via a superacid-catalyzed reaction in an organic sol-gel way [51]. The membrane channel geometry is adjusted by deploying monomers of different rigidity and the channel chemistry is regulated by varying the functional groups with negative or positive charges. As a result, the optimum CTF membranes possess rigid and charged channels at the ultra-microporous scale (<0.7 nm) and multiple channel chemistries (Fig. 5f).

### Ion transport behaviors under a microporous confinement regime

The microporous confinement region of new-generation IEMs can provide a confined space to boost the interactions (i.e. electrostatic interaction, hydrogen bonding interaction, and coordination interaction) between ions and channel walls, generating significant impacts on ion transport behaviors. The size of the micropores can be modulated from sub-2-nm to sub-1-nm and even under 0.7 nm, affecting the arrangement and distribution of the ion hydration shells and inducing ion dehydration, thereby further enhancing the interaction between dehydrated ions and the functional sites on the channel wall, thus endowing fast and selective ion transport. Ion transport properties in the sub-2-nm channels, sub-1-nm channels, and ultra-microporous channels are illustrated to show the microporous confinement effects of new-generation IEMs.

**Figure 6.**
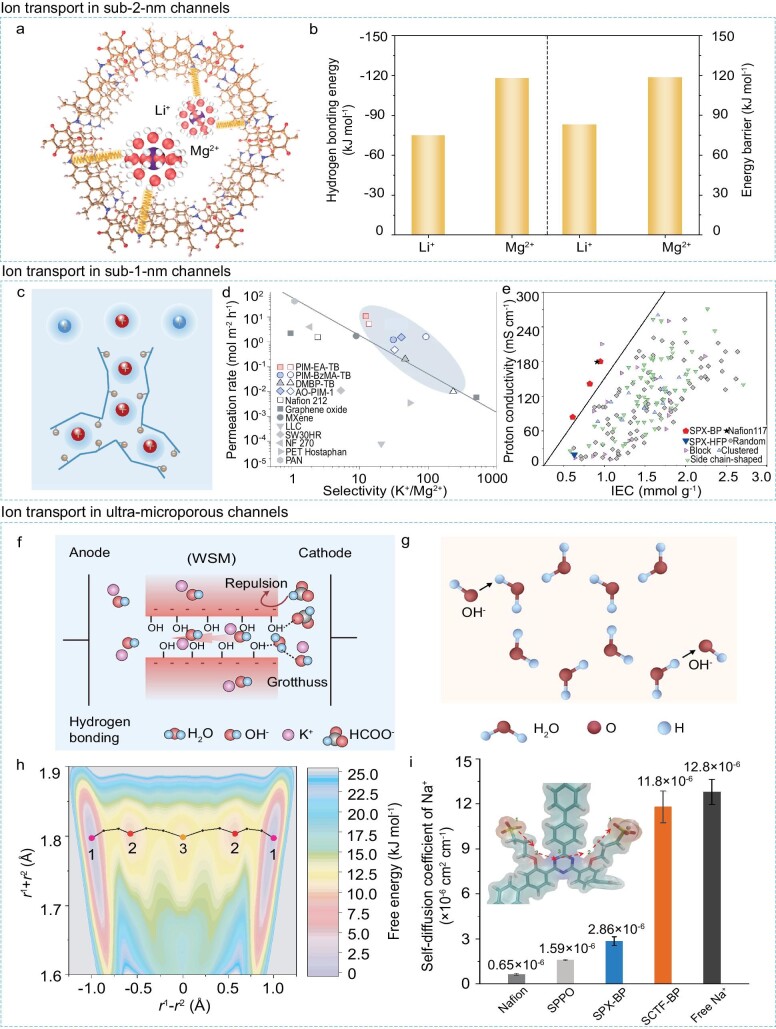
Atypical ion transport properties under a microporous confinement regime. (a) Hydrogen bond interaction between hydrated ions and the COF channel. (b) Hydrogen bonding energy and energy barrier for ion transport. Adapted from Ref [52], John Wiley and Sons. (c) Selective ion transport in the negatively charged PIM channels. (d) Comparison of ion permeation rate and selectivity of PIM membranes with other IEMs. Adapted from Ref [54], Springer Nature Limited. (e) Comparison of proton conductivity and IEC of PIM membranes with traditional IEMs. Adapted from Ref [36], John Wiley and Sons. (f) The formation of continuous hydrogen-bonding networks in micropore confinement channels. (g) The fast transport of OH^−^ through the Grotthuss mechanism. Adapted from Ref [58], Springer Nature Limited. (h) Ion transport in the CTF channels via multiple-interactions. (i) Comparison of Na^+^ diffusion coefficient of CTF membranes with other IEMs. A near-frictionless ion transport through the CTF membrane. Adapted from Ref [51], Springer Nature Limited.

The channel sizes of COF membranes are larger than the diameter of hydrated ions, thus, ions can transport through the channels without dehydration. Hydrogen bonds can be formed between water molecules in the hydration shells of hydrated ions and the hydrogen bonding sites on the COF channel wall. The hydrogen bonding energy varies with ion charge density, where multivalent ions generate a higher bonding energy than monovalent ions. Since hydrated Li^+^ and Mg^2+^ ions are surrounded by water molecules, the hydrogen bonds can form between hydrated ions and the channel wall [52] (Fig. 6a). The hydrogen bonding energy of Li^+^ is lower than that of Mg^2+^, leading to a lower energy barrier for Li^+^ transport than that of Mg^2+^ (Fig. 6b). In addition, the solvation/coordination interactions between the ions and the channel wall are deployed to regulate ion transport behaviors [53]. Oligoether chains with varying lengths of ethylene oxide (EO) units are introduced in the COF channels. The solvation capabilities of lithiophilic oligoethers vary with the numbers of EO units, since EO units can substitute the water oxygen atoms surrounding the ions. The energy barrier differences between ion transport through the channels can be adjusted by the length of EO within the channels. The optimum length of oligoether is two EO units, which shows the largest difference between the energy barrier of Li^+^ and Mg^2+^. The SBP membranes are positively charged with a loose chain packing to facilitate the transport of anions (i.e. Cl^−^) with a low energy barrier in the sub-1-nm channels. Thus, the microporous anion exchange membranes show an ultrahigh Cl^−^ conductivity (∼60 mS cm^−1^ at 30°C and ∼120 mS cm^−1^ at 80°C), surpassing those of both traditional and other commercial IEMs.

For PIMs of sub-1-nm channels, ions should be partially dehydrated to transport across the membrane. The monovalent ions of smaller sizes can be sieved from the multivalent ones of larger sizes, considering the higher ion dehydration energy required for multivalent ions than monovalent ones. Upon partial dehydration, the shielding effect from the hydration shells is weakened so as to enhance the electrostatic interactions between ions and the surface charge on the channel wall (Fig. 6c). For instance, the electrostatic attraction toward cations of a negatively charged PIM is dramatically boosted, facilitating the fast transport of cations and leading to a high mono/multivalent cation selectivity. Therefore, PIM-IEMs show a fast and selective transport of monovalent ions by the enhanced size sieving and electrostatic interactions. Fast cation transport within negatively charged PIM membranes were fabricated based on the typical PIM-1 via the reaction between nitriles and hydroxylamine, obtaining amidoxime group-containing PIMs (AO-PIM) [54,55]. Amidoxime groups became negatively charged in strong alkaline aqueous environments, producing AO-PIM membranes combining high ion conductivity and high membrane selectivity (Fig. 6d). It should be noted that PIM membranes using amidoxime as functional groups tend to lose their high performance in conducting cations at low pH. This is because amidoxime maintains a negatively charged state only when the pH is above 13 [56]. This issue can be addressed by PIM membranes using sulfonic groups as functionality, namely sulfonated polyxanthene (SPX) [36]. SPX membranes had an obvious advantage over Nafion membranes in terms of balancing well with the ion conductivity and selectivity (Fig. 6e). The SPX membranes all had IECs below 1 mmol/g, and increasing the content of charged groups seems to be a simple way to pursue higher ion conductivity. C. Ye *et al.* reported sulfonated spirobifluorene-containing PIMs (sPIM-SBF) with IECs ranging from 0.53 to 1.86 mmol g^−1^, which were synthesized from sulfonating dibenzodioxane-based ladder polymers with trimethylsilyl chlorosulfonate [57]. As expected, increasing IECs significantly improved the membrane conductivity which finally reached a very high value of 39.4 mS cm^−1^ (11.9 mS cm^−1^ for Nafion 115 under the same testing condition), thus leading to decreased membrane resistance in AORFBs. However, sPIM-SBF membranes with increased IECs from 0.53 to 1.86 mmol g^−1^ were found to present a much decreased membrane selectivity, with K_4_Fe(CN)_6_ permeabilities dropping dramatically from 1.1 × 10^−12^ to 7.5 × 10^−9^ cm^2^ s^−1^. This decreased selectivity is most likely due to the severe water uptake-induced swelling of membranes. The PIM-SBF membrane with an IEC of 1.86 mmol/g exhibited a very high swelling ratio of 32%.

IEMs with ultra-micropores can render ion dehydration to a larger extent, which contributes to the original ion's property of hydrated ions. Micropore confinement can contribute to the formation of continuous hydrogen-bonding networks, which are beneficial for facilitating the transport of OH^−^ via the Grotthuss mechanism [58]. Moreover, the positively charged groups in the microporous channels enable the exchange of OH^−^ via the vehicle mechanism (Fig. 6f and g). Hence, the well interconnected and narrowly distributed ultra-micropores in MTCP membranes enable the fast transport of OH^−^ through the Grotthuss and vehicle conduction. The MTCP-50 membrane exhibits a high OH^−^ conductivity of 78.4 mS cm^−1^ at 30°C and drastically increases to 217.0 mS cm^−1^ with the temperature rising to 90°C, which is far beyond those of the traditional AEMs [34]. The charged, polar and acidic segments in the CTF framework channels allow multiple interplays involving the electrostatic, dielectric, and coordination interactions between monovalent cations and the channel wall. More importantly, the higher dehydration energy barriers for ion transport in the ultra-microporous channels can be compensated by the multiple ion-channel interactions. The rigidity-confined ion channels accompanied by the manifold interactions deliver a low energy barrier for monovalent cation transport, realizing a near-frictionless ion flow through the CTF membrane with a high ion selectivity [51] (Fig. 6h and i).

## APPLICATIONS

New-generation IEMs allow ion transport under a microporous confinement regime and bring about atypical ion transport behaviors, generating a high ion permeability/conductivity and selectivity superior to traditional IEMs, thus enabling outstanding performance in applications including ion separation, flow battery, water electrolysis, and ammonia synthesis. For example, COF membranes provide ordered ion transport channels, and the intensified interaction between ions and the channel wall can differentiate the ion transport rates and thus induce a selectivity for ion separation. CTF membranes with ultra-micropores and multiple interactions within the channels enable a low ion transport barrier and a high selectivity for ions over active electrolytes, showing great potential in aqueous flow batteries. The MTCP are ultra-microporous anion exchange membranes with positively charged sites confined in the ultra-micropores, facilitating fast OH^−^ transport by the reinforced electrostatic attraction in the channels. The high alkaline-resistance and OH^−^ conductivity make the MTCP membranes a competitive candidate for water electrolysis to produce hydrogen. The highly OH^−^ conductive MTCP membranes can also contribute to electrochemical ammonia synthesis. Therefore, the representative applications of new-generation IEMs are introduced.

### Li^+^ ion separation

**Figure 7.**
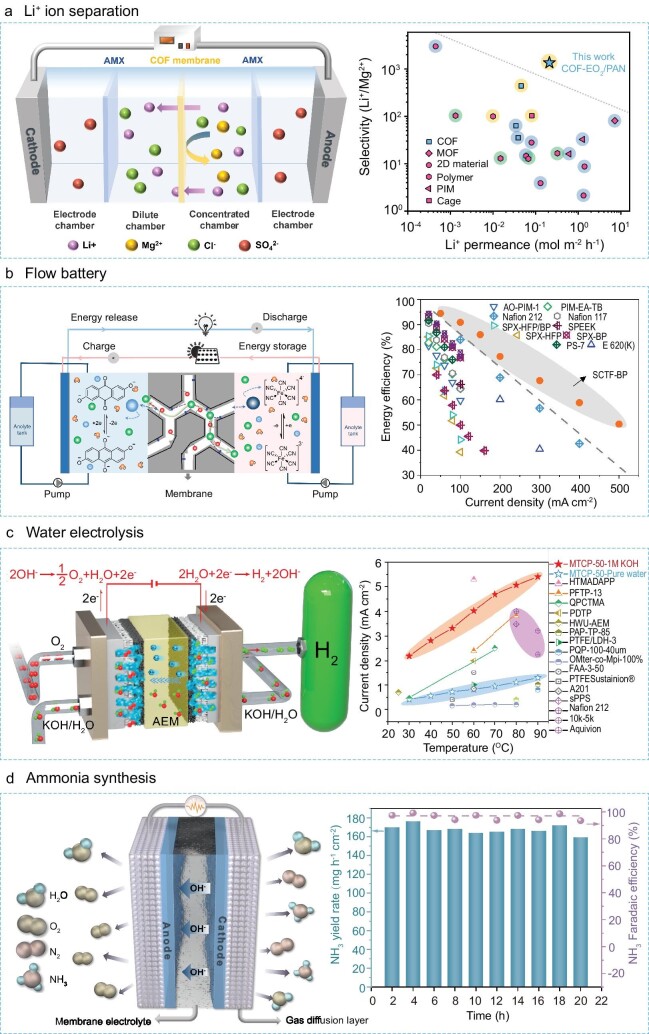
Applications of new-generation IEMs. (a) COF membranes for Li^+^ ion selective separation via an electrodialysis process. The separation performance of COF membranes is better than that of traditional IEMs and other reported membranes. Adapted from Ref [53], the National Academy of Sciences. (b) CTF membranes for AORFBs. The energy efficiency at varied current densities of CTF membranes are superior to other typical membranes. Adapted from Ref [51], Springer Nature Limited. (c) MTCP-50 membranes for water electrolysis. The current densities of the MTCP-50 membrane at varied temperatures are higher than traditional IEMs (MTCP-50 fed with 1 M KOH at 1.8 V). Adapted from Ref [34], Springer Nature Limited. (d) MTCP-50 membranes for electrochemical NH_3_ synthesis. NH_3_ yield rate and the corresponding Faradaic efficiency versus time. Adapted from Ref [66], John Wiley and Sons.

The minable lithium resources in salt lakes account for 60%–80% of the world’s production. Lithium-ion selective membranes play an important role in extracting lithium from salt lakes, so as to guarantee the rapidly growing demand for lithium from the lithium-ion battery industry [59]. The as-prepared COF membranes are assembled into an electrodialysis cell, and the Li^+^/Mg^2+^ separation performance is evaluated under the electro-driven conditions. Under sub-2-nm confinement, the enhanced interaction between hydrated ions and the COF channel wall can differentiate the ion transport rates, realizing a selectivity for monovalent over multivalent ions based on the interaction screening effect. As a result, Li^+^ shows a permeation rate of ∼0.2 mol m^−2^ h^−1^, higher than that of Mg^2+^, and the COF membrane exhibits a Li^+^/Mg^2+^ selectivity of ∼1300 in the binary-salt system (Fig. 7a). The Li^+^/Mg^2+^ separation performance of COF membranes surpasses traditional IEMs and breaks the trade-off limitation owing to the confinement effect [53], which demonstrate a great potential in the application of lithium extraction from salt lakes.

### Flow battery

New clean energies from nature, such as solar and wind energy, feature intrinsic intermittency and thus their efficient utilization requires safe, cost-effective storage technologies that enable on-demand integration of new energies into power grids [60]. Aqueous organic redox flow batteries (AORFBs) that use the reversible redox reactions of redox-active species dissolving in aqueous solutions to store electricity exhibit advantages such as nonflammability, cost efficiency, decoupled energy and power, and long duration, and therefore have attracted substantial attention worldwide [61]. Within AORFBs, an IEM is required to conduct charge-carrying ions and simultaneously avoid the crossover of redox-active species, and its capacity to fulfill selective ion conduction largely determines the efficacy of AORFBs [62]. The emerging microporous membranes, especially ultra-micropore confined IEMs, combine high free volume-induced permeability with high micropore confinement-imposed size selectivity, thus endowing AORFBs with both high energy efficiency at high current densities and extremely low-capacity loss induced by crossover of actives. For example, triazine framework membranes used in alkaline anthraquinone/K_4_Fe(CN)_6_ AORFBs exhibited an extremely low area-specific membrane resistance of 0.17 Ω cm^2^, and thus enabled fast-charging and stale-cycling AORFBs that presented both high energy efficiency and high capacity utilization at varied current densities up to 500 mA cm^–2^ (Fig. 7b) [51]. This performance far surpasses those for otherwise identical AORFBs assembled with typical reported membranes.

### Water electrolysis

Water electrolysis is a carbon-free technology for producing hydrogen, where AEMs with high alkaline stability and OH^−^ conductivity play an essential role [63]. The MTCP-50 membrane shows a current density of ∼2.2 A cm^−2^ at 1.8V and 30°C when fed with 1 M KOH solution, and a higher current density of ∼5.4 A cm^−2^ can be achieved at 90°C. The high energy output performance can be attributed to the fast transport of OH^−^ through the ultra-microporous AEM, enhancing the mass transfer efficiency of the electrolyzer from anode to cathode. From the comparison of the current densities of membrane-based water electrolysis, the MTCP-50 membrane displays a higher performance than those of reported AEMs (e.g. <2.0 A cm^−2^), and superior to commercial PEM water electrolysis (e.g. ∼3.0 A cm^−2^). The MTCP-50 membrane-based water electrolyzer exhibits a long-term durability in 1 M KOH, which is operated at a current density of 0.5 A cm^−2^ at 60°C, showing a 0.015 mV h^−1^ voltage decline rate during 2500 h. Moreover, MTCP-50 membrane-based water electrolysis can be performed at a current density of 1.0 A cm^−2^ at 80°C for 500 h with a decay rate of 0.13 mV h^−1^ (Fig. 7c) [34]. The ultra-microporous MTCP-50 membrane exceeds the state-of-the art AEMs in terms of high current density and long-term durability, demonstrating great potential in the application of water electrolysis.

### Ammonia synthesis

Ammonia (NH_3_), as a chemical compound and energy carrier, is essential for modern agriculture and industry [64]. The Haber-Bosch process involves the reaction between N_2_ and H_2_ at high temperature and pressure. As an alternative, the low-temperature electrochemical NH_3_ synthesis from N_2_ and H_2_O driven by sustainable energy has recently attracted much attention [65]. AEMs are crucial components of the electro-synthesis devices to transport OH^−^ and separate electrolytes on both sides and prevent the cross diffusion of NH_3_. The ultra-microporous MTCP-50 membranes were also assembled in the H-type electrolysis cell for the electrochemical synthesis of NH_3_. The Faradaic efficiency (FE) exhibits a high value of 98.1% at −0.7 V, followed with a record-high NH_3_ yield rate of 164.3 mg h^−1^ cm^−2^, surpassing the F_NH3_ (87.5%) and NH_3_ yield (144 mg h^−1^ cm^−2^) achieved by traditional IEMs (Nafion). Moreover, no obvious decline is observed in the FE and NH_3_ yield rate during long-term electrolysis, confirming the stability of the MTCP-50 membrane in the NH_3_ synthesis system (Fig. 7d) [66]. The NH_3_ yield can reach 4.8 g h^−1^ after 1-h electrolysis by further increasing the electrolytic cell working area. This suggests the huge potential application of ultra-microporous AEMs in the electrochemical synthesis of NH_3_.

## OUTLOOK AND PERSPECTIVE

The new-generation IEMs featuring microporosity, rigid frameworks, and ordered ion transport channels can establish a micropore-confined space to significantly boost the size and interaction effects on ion transport and sieving properties. Despite the great potential of new-generation IEMs, future development in terms of intricate microstructure observation, *in-situ* ion transport visualization, and large-scale membrane fabrication deserves more attention and in-depth exploration.

First, the microporous structure of new-generation IEMs is indistinct. The channel size is normally reflected by the pore size distribution determined via the sorption isotherms or positron annihilation spectrum, which gives statistical results. The channel geometry is generally deduced from a theoretical model ignoring the effects of the membrane synthesis condition and applied external field. Moreover, there is still a knowledge gap in the distribution of the functional sites along the channels, and the distance between functional groups may greatly affect ion transport properties [67]. Thus, the characterizations of the precise channel configuration and the pinpoint location of functional sites require direct observation by microscopy techniques at the angstrom scale, which can further expand the scope of building blocks (e.g. 2D materials, crystalline framework materials) [68] for the next-generation IEMs.

Second, the ion transport trajectory in the confined microporous framework channels and the interaction between ions and the channel wall is mostly indicated via molecular dynamic simulations and calculated by density function theory, respectively. Pulsed-field gradient NMR has been employed to determine the local ion diffusion coefficients in the framework channels, which can only reveal the specific ion transport behaviors in membranes. Time of flight mass spectrum has been adopted to demonstrate the ion dehydration process by comparing the hydrated water molecules around ions before and after passing through membranes of sub-1-nm channels, but the experience of the ion dehydration-rehydration process within the membranes still remains unknown. Therefore, *in-situ* monitoring techniques are required to visualize the ion transport tracks and figure out the multi-interplays between ions and interaction sites, so as to build ion transport models and achieve a frictionless ion transport in membranes.

Last but not least, the large-scale fabrication of microporous framework membranes is of significant importance but is challenging. Microporous framework membranes aim at the separation of ions at the sub-nm scale, where defect-free membranes are required, since any defects in the framework or between the frameworks can ruin ion selectivity. In addition, a majority of microporous framework materials are devoid of solution processibility, rendering it difficult to prepare the microporous framework membranes through the solution casting method. Tuning the polymerization degree to obtain soluble microporous framework materials or applying the organic sol-gel method can be effective strategies to fabricate large-scale membranes, which is worth an in-depth exploration to establish a universal guideline for a broad range of microporous framework materials. Uniform and defect-free new-generation IEMs at a large-scale can contribute to the wide application in the fields of precise ion separation, clean energy production, and renewable energy storage and conversion.
